# Influence of Vanadium Micro-Alloying on the Microstructure of Structural High Strength Steels Welded Joints

**DOI:** 10.3390/ma16072897

**Published:** 2023-04-05

**Authors:** Giulia Stornelli, Anastasiya Tselikova, Daniele Mirabile Gattia, Michelangelo Mortello, Rolf Schmidt, Mirko Sgambetterra, Claudio Testani, Guido Zucca, Andrea Di Schino

**Affiliations:** 1 Dipartimento di Ingegneria, Università degli Studi di Perugia, Via G. Duranti 93, 06125 Perugia, Italy; andrea.dischino@unipg.it; 2 Vantage Alloys AG, 6300 Zug, Switzerland; anastasiya.tselikova@vantage-alloys.com (A.T.); rolf.schmidt@vantage-alloys.com (R.S.); 3 Dipartimento Sostenibilità dei Sistemi Produttivi e Territoriali, ENEA—CR Casaccia, 00123 Rome, Italy; daniele.mirabile@enea.it; 4 Sede di Genova, Istituto Italiano della Saldatura, Lungobisagno Istria 15, 16141 Genova, Italy; michelangelo.mortello@iis.it; 5 Aeronautical and Space Test Division, Italian Air Force, Via Pratica di Mare 45, 00040 Pomezia, Italy; mirko.sgambetterra@gmail.com (M.S.); zucca.guido@gmail.com (G.Z.); 6 CALEF-ENEA CR Casaccia, Via Anguillarese 301, Santa Maria di Galeria, 00123 Rome, Italy; claudio.testani@consorziocalef.it

**Keywords:** vanadium micro-alloying, microstructure, residual austenite, precipitation

## Abstract

The inter-critically reheated grain coarsened heat affected zone (IC GC HAZ) has been reported as one of the most brittle section of high-strength low-alloy (HSLA) steels welds. The presence of micro-alloying elements in HSLA steels induces the formation of microstructural constituents, capable to improve the mechanical performance of welded joints. Following double welding thermal cycle, with second peak temperature in the range between Ac1 and Ac3, the IC GC HAZ undergoes a strong loss of toughness and fatigue resistance, mainly caused by the formation of residual austenite (RA). The present study aims to investigate the behavior of IC GC HAZ of a S355 steel grade, with the addition of different vanadium contents. The influence of vanadium micro-alloying on the microstructural variation, RA fraction formation and precipitation state of samples subjected to thermal cycles experienced during double-pass welding was reported. Double-pass welding thermal cycles were reproduced by heat treatment using a dilatometer at five different maximum temperatures of the secondary peak in the inter-critical area, from 720 °C to 790 °C. Although after the heat treatment it appears that the addition of V favors the formation of residual austenite, the amount of residual austenite formed is not significant for inducing detrimental effects (from the EBSD analysis the values are always less than 0.6%). Moreover, the precipitation state for the variant with 0.1 wt.% of V (high content) showed the presence of vanadium rich precipitates with size smaller than 60 nm of which, more than 50% are smaller than 15 nm.

## 1. Introduction

Recent developments of high strength low alloyed steels (HSLA) for energy sector focused on the need to target optimized combinations of strength, toughness, weldability on industrial scale at affordable prices [1,2,3,4,5]. A similar scenario also applies over other application sectors (e.g., offshore structural application and shipbuilding) with different specific requirements, which are set as a function of technological and exercise needs [6]. Vanadium, due to its own thermodynamic and kinetic ability to precipitate in form of carbide and nitride, is considered a key element in the metallurgical design of modern HSLA steels [7,8,9], as it enables efficient and cost-effective solution across a broad range of applications [10,11,12,13]. For example, in high strength-high toughness steels for pipelines, the increase in the available strength level up to X80–X100 grade of line pipe steels, promoted in the latest decades, has produced economic advantages estimated in the billion-dollar range [14].

The evolution of the effect of microalloying on the microstructure and the properties of a girth weld is challenging as this depends on the number of inter-correlated metallurgical phenomena correlated to the steel chemical composition and the welding processing conditions [15,16,17].

Despite the importance of microalloying for the development of high strength steels with increased toughness, a decay of material properties in girth welded joints is reported in literature [18] which has limited an excessive use of microalloying.

Thermal cycles experienced during welding have a large impact on equilibrium set between high strength and high toughness in HSLA steels as these cycles are the main cause of toughness loss in the heat affected zone (HAZ).

Welds and heat-affected zones are critical when considering structural integrity, specifically, fracture and fatigue properties [19,20]. Weld design in thicker gauge plates requires consideration of the time required to perform the weld, which is partially controlled through the heat input. With the aim of saving time, reducing component manufacturing costs, and improving efficiency, high heat input welding technology has been widely used.

Historically, the lowest toughness was expected in the grain coarsened heat affected zone (GC HAZ), which is the part of the HAZ closest to the welding fusion line [21,22,23,24]. During welding, the GC HAZ experiences peak temperatures up to the melting point, followed by rapid cooling. The high temperatures can lead to significant austenite grain coarsening [25], the combination of a coarse austenite grain size and rapid cooling promotes brittle microstructures, which contain high proportions of ferrite side-plates and bainite [26].

In recent years, it has been found that the most degraded part in the HAZ is the inter-critically reheated grain coarsened HAZ (IC GC HAZ), which is the region of the GC HAZ reheated to temperatures between the Ac1 and Ac3 by subsequent welding passes [27]. During the inter-critical thermal cycle, partial transformation to austenite occurs, particularly where austenite stabilizers, such as carbon or manganese, are segregated in the initial microstructure [28]. These areas include pearlite/bainite colonies. When cooling, these high carbon regions transform into pearlite/bainite or residual austenite (RA) depending on the hardenability of the austenite and cooling rate [29]. The presence of RA phase is generally regarded as the major factor which reduces the HAZ toughness [30,31].

However, it is also reported that the loss in toughness is not just due to the presence of RA phase, but is related to the distribution and morphology of the RA constituent, and the matrix microstructure. Cui et al. [32] reported that block residual austenite significantly deteriorates impact toughness of super-critical reheated coarse grain heat affected zone (SC CGHAZ).

Niobium is commonly added to enhance the strength of HSLA steels. However, under welding conditions, niobium adoption shows detrimental effect on the HAZ toughness, although its effect is strongly dependent on heat input [33]. At medium to high heat input despite a precipitation hardening effect via Nb(C,N), niobium has a detrimental influence on the fracture toughness of GC HAZ. Niobium reduces the grain boundary ferrite formation and promotes the nucleation of coarse structure of ferrite with aligned RA resulting in increased mechanical properties. A small addition of niobium suppresses ferrite nucleation at prior austenite grain boundaries and increase the volume fraction of either martensite or bainite. Previous studies reported that the major advantages of a niobium addition, i.e., the grain refinement and the resultant improvement of base metal mechanical properties, appear to be outweighed by the detrimental effects of martensite formation, when the steel plates are welded [34].

On the other hand, vanadium leads to grain refinement and precipitation strengthening to HSLA steels. The effect of vanadium on the GC HAZ microstructure is quite different from that of niobium. Vanadium has a beneficial effect on the toughness of the GC HAZ, because it reduces the bainitic colony size and, due to the low misfit between vanadium nitrides (VN) and ferrite in comparison with other types of inclusions, promotes intragranular nucleation of acicular ferrite [20,35].

Moreover, alloying associated with the precipitate formation is an important consideration in weld design to achieve desirable microstructures in the HAZ [36]. For instance, Zajac et al. [37] performed HAZ simulations on 25 mm-thick HSLA plates (Fe-0.09%C-1.4%Mn-0.08%V-0.010%Ti-xN) with low (0.003%N) and high (0.013%N) nitrogen contents. They showed that for the high nitrogen steel, intragranular forms for a wider range of cooling rates as compared to the low nitrogen steel. In contrast, in high nitrogen steel for the high heat input (slowly cooled) conditions, a significant fraction of coarse ferrite grains forms at the austenite grain boundaries, which leads to poorer toughness compared to the low nitrogen steel. Zajac et al. [37] interpreted that the increased fraction of coarse grain boundary ferrite was associated with V(C,N), the amount of which would likely be increased during slow cooling; it should be noted that the mechanism for V(C,N) to accelerate the formation of grain boundary ferrite is not clearly discussed in the study. Zajac et al. [37] also pointed out that the precipitation status, which depends on the peak temperature in HAZ simulation, influences austenite grain size and amount of ‘free’ nitrogen, both of which affect phase transformations upon cooling.

Hu et al. [38] and Wu et al. [39] showed that V(C,N) with sizes between 20–30 nm were not detrimental on impact toughness because of their small sizes.

Following the above mentioned latest developments, the influence of vanadium on the toughness and fatigue resistance of the IC GC HAZ is not fully understood and requires further investigation. To understand the effective resistive behavior due to the addition of vanadium in the IC GC ZTA of a welded joint, it is first of all considered appropriate to investigate the real effect of the alloying elements on the microstructural characteristics.

In this regard, this study aims to assess the effect of vanadium alloying on material properties (in terms of microstructural constituent variation, RA formation and precipitation state) of an S355 steel (EN10025-2), when subjected to welding-representative thermal cycles in the IC GC HAZ.

## 2. Materials and Methods

S355 steel grade (EN10025-2) plates for structural application were manufactured by Vacuum Induction Melting (VIM) plant in the form of three 80 kg ingots (diameter 120 mm) in four variants, including its base reference. The nominal chemical composition of the considered steels are reported in Table 1.

The ingots have been hot rolled down to 16 mm thickness in 10 passes. The steels chemical compositions to be investigated were designed in order to have a Carbon Equivalent Content (Ceq) value lower than 0.42%, according to International Institute of Welding (IIW) Equation (1), as a function of weight percentage (%) [40]:

(1) $$\mathrm{Ceq}=\%\;C+\frac{\%\;\mathrm{Mn}\;}{6}+\frac{\%\;\mathrm{Cr}+\%\;\mathrm{Mo}+\%\;V}{5}+\frac{\%\;\mathrm{Cu}+\%\;\mathrm{Ni}}{15}$$

**Figure 1:**
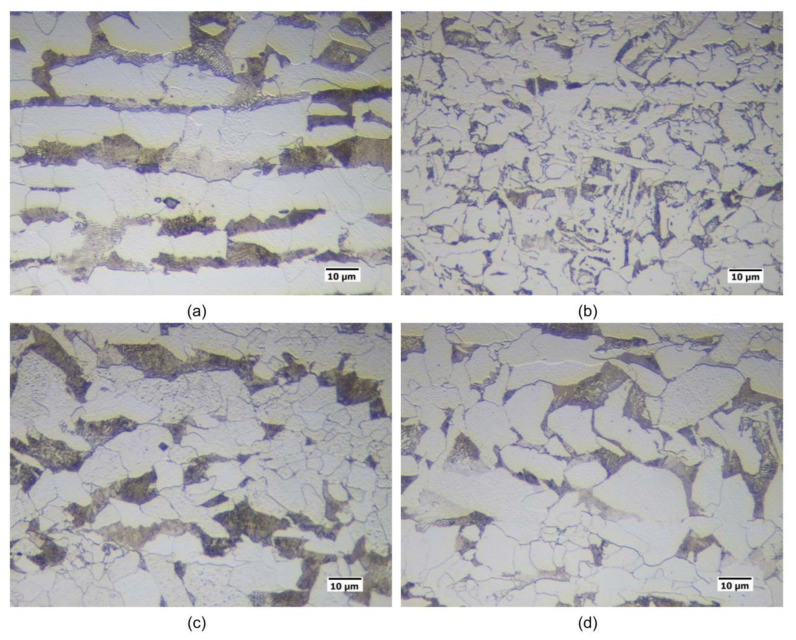
Hot rolled material (2% Nital etching) ((**a**) Reference material, (**b**) Variant I, (**c**) Variant II, (**d**) Variant III).

**Figure 2:**
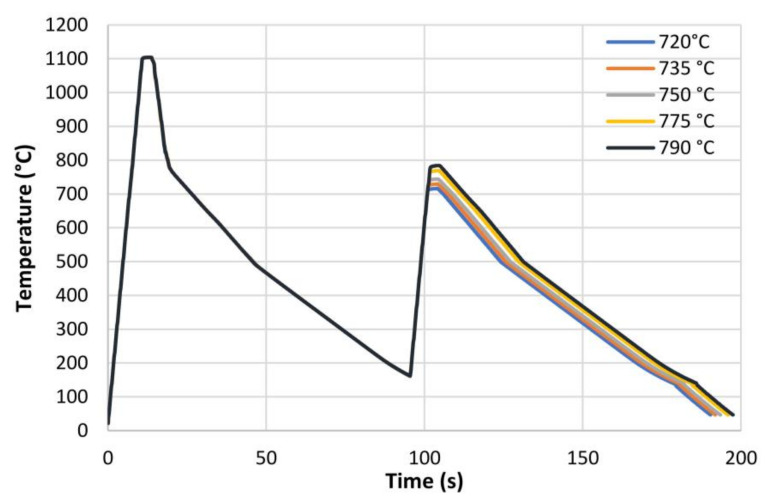
Experimental thermal profiles as acquired by thermocouples as obtained by dilatometry.

The hot rolled microstructures are reported in Figure 1 after 2% Nital etching. Starting from the hot rolled material, cylindrical specimens (10 mm in length, 4 mm in diameter) were machined to be heat treated in controlled conditions by using a dilatometer. The IC GC HAZ thermal cycles, in accordance with Figure 2, were designed to simulate a double pass submerged arc welding process with heat input of 2.5 kJ/mm in 16 mm thick plate [41]. The initial temperature of the first pass was assumed at the room temperature (25 °C) while, for the second pass, the value was set to 150 °C. Because of the technological limitations of the dilatometer, the samples were heated up to 1100 °C with a heating rate of 100 °C/s whilst the holding time was set at 3 s. The cooling profile was set in order to guarantee the cooling time between 800 °C and 500 °C (t8/5) of about 25 s [41,42]. The second peak of weld conditions, with a peak temperature in the inter-critical zone, was selected by considering the values of critical temperature Ac1 and Ac3. In fact, these temperatures, obtained by dilatometric test, were reported in Table 2 and they are dependent on steel variants. Ac1 and Ac3 can be estimated through empirical equations taking into account the alloy elements [43]. However, in this study Ac1 and Ac3 were assumed equal to 715 °C and 815 °C respectively so that the peak temperature of the second pass was in the inter-critical zone for all steel variants. Therefore, the heat treatments were designed with the aim to reproduce different microstructures corresponding to different positions of the HAZ in a welded joint. In particular, the inter-critical zone of the second welding pass has been simulated with five different peak temperatures: 720, 735, 750, 775 and 790 °C (see Figure 2).

In order to investigate the presence of residual austenite (RA) and to define the most suitable methodology to assess the RA presence in the considered steels after the heat treatment, three different methods have been applied: X-ray Diffraction (XRD), Electron Backscattered Diffraction (EBSD), and LePerà selective etching. XRD analysis was carried out by using a Smartlab Rigaku diffractometer equipped with Cu kα source radiation and a D/teX Ultra 250 SL detector, operated at 40 kV and 30 mA in continuous mode in the angular range 30–110 2θ degree. The automated sample alignment routine has been used. EBSD measurements were performed with the aim to detect the presence and position of RA islands, by means of a field emission gun scanning electron microscope (FEG-SEM) (Ultra-Plus Carl-Zeiss-Oberkochen, Jena, Germany) equipped with an EBSD detector (C Nano Oxford Instruments, Stockholm, Sweden), using a 0.1 μm scanning step size. RA was revealed by building up phase maps, taking into account both face-centered cube (fcc) and body-centered cube (bcc) phases: automatic image analysis of such maps allowed to determine RA volume fraction.

LePerà solution (1 g H_2_S_2_O_5_ + 100 mL H_2_O + 4 g C_6_H_3_N_3_O_7_ + 100 mL C_2_H_5_OH) for about 60–90 s was conducted for selective etching. The microstructure was then analyzed by optical microscopy (OM) (Eclipse LV150 NL, Nikon, Tokyo, Japan) whilst the image analysis was performed using dedicated software (AlexaSoft, X-Plus, serial number: 6308919690486393, Florence, Italy), in order to determine the RA fraction. The procedures were performed on low magnification image and on three different fields: the RA % reported refers to the average of three values. Vickers hardness tests were made by means of a HV50 (Remet, Bologna, Italy) instrument by using a load of 10 kg. Three hardness tests were performed on each sample. Precipitation state was analyzed by transmission electron microscope (TEM) on extraction replica specimens. The observations were performed with a JEOL 200CX transmission electron microscopy (JEOL Ltd., Tokyo, Japan). The analysis was carried out over a significant area, evaluating the chemical composition (by means of EDX analysis) and the average size of the precipitates, within a limit of 50 precipitates for each sample analysed.

## 3. Results and Discussions

### 3.1. Microstructure and Hardness

**Figure 3:**
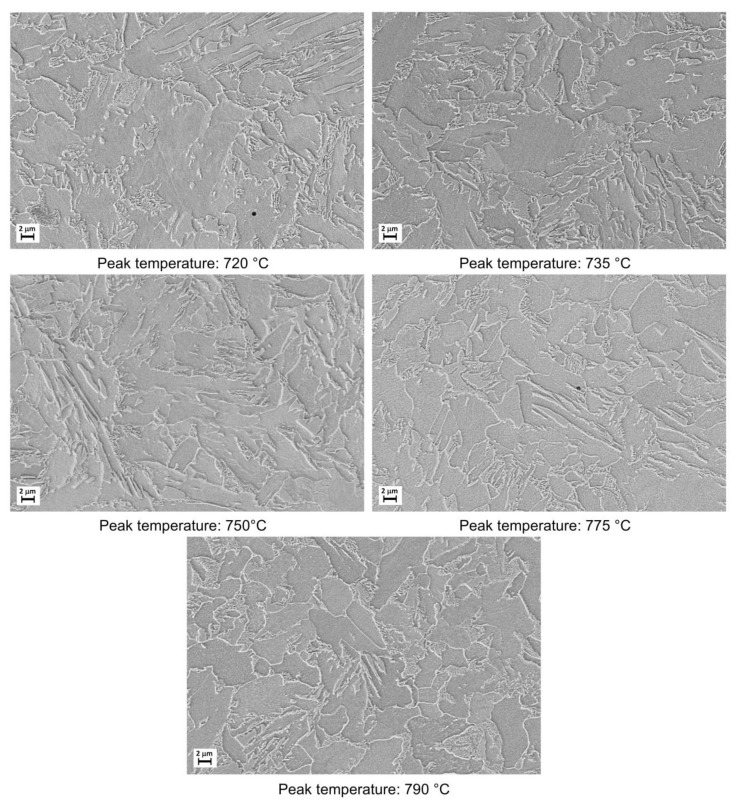
Microstructures as obtained by dilatometric cycles (reference material).

**Figure 4:**
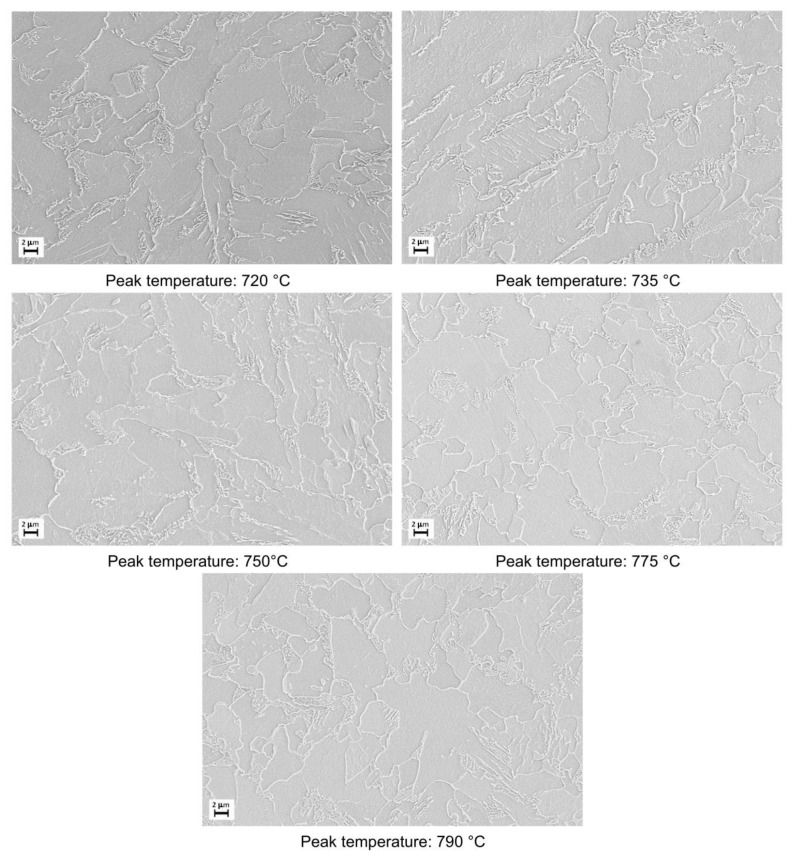
Microstructures as obtained by dilatometric cycles (variant I).

**Figure 5:**
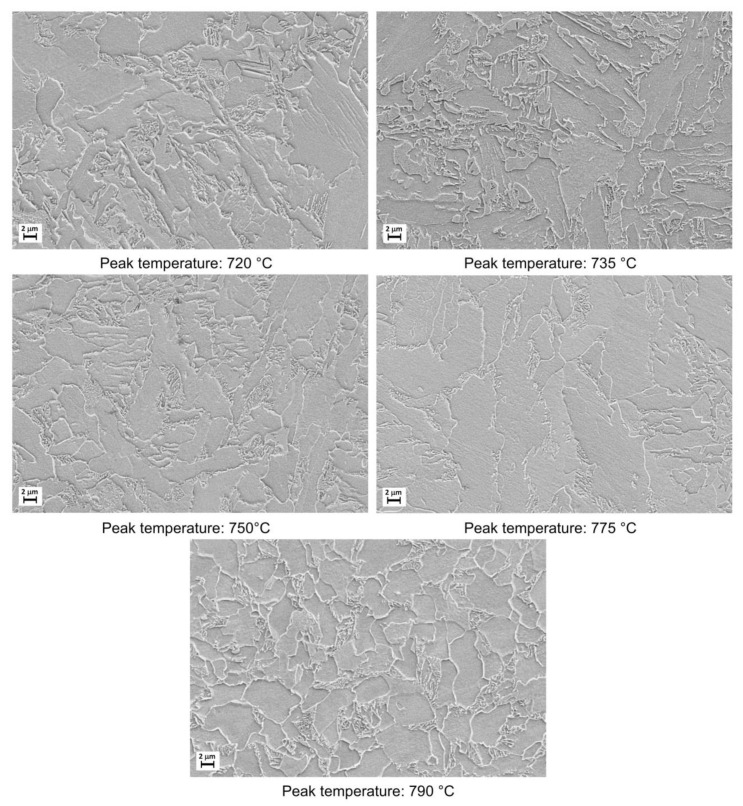
Microstructures as obtained by dilatometric cycles (variant II).

**Figure 6:**
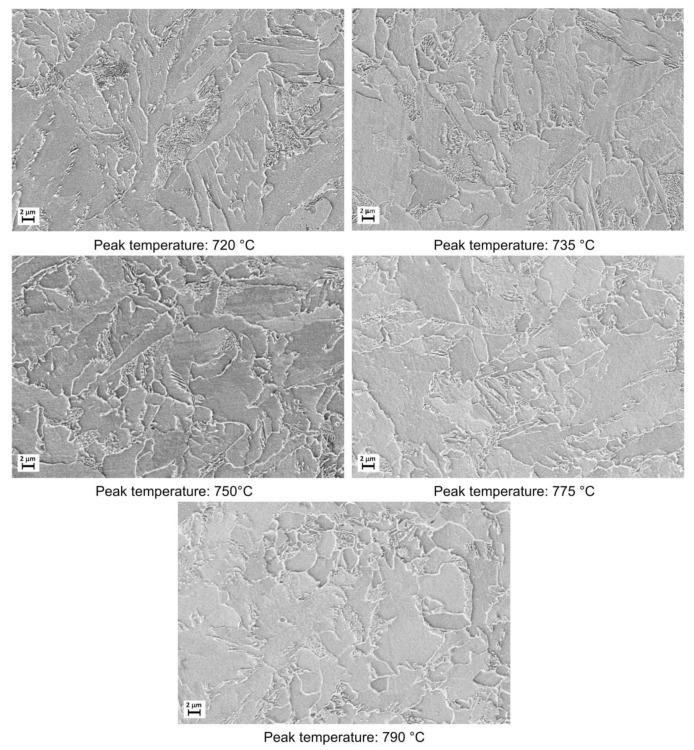
Microstructures as obtained by dilatometric cycles (variant III).

The microstructural evolution detected by SEM for all the considered steels subjected to heat treatments (as shown in Figure 2) is reported in Figure 3, Figure 4, Figure 5 and Figure 6.

**Figure 7:**
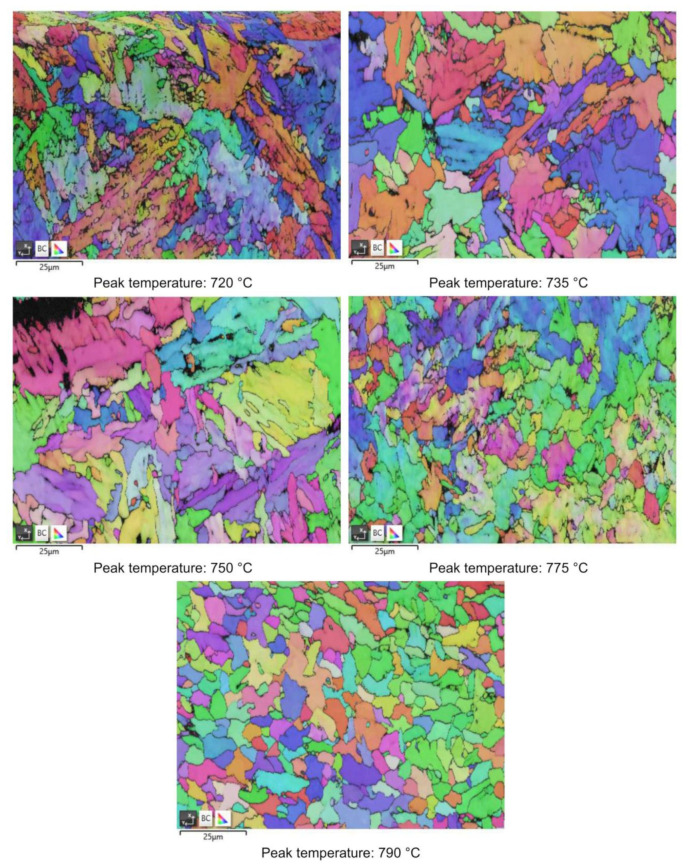
EBSD polar figure maps of specimens subjected to dilatometric cycles (reference material).

**Figure 8:**
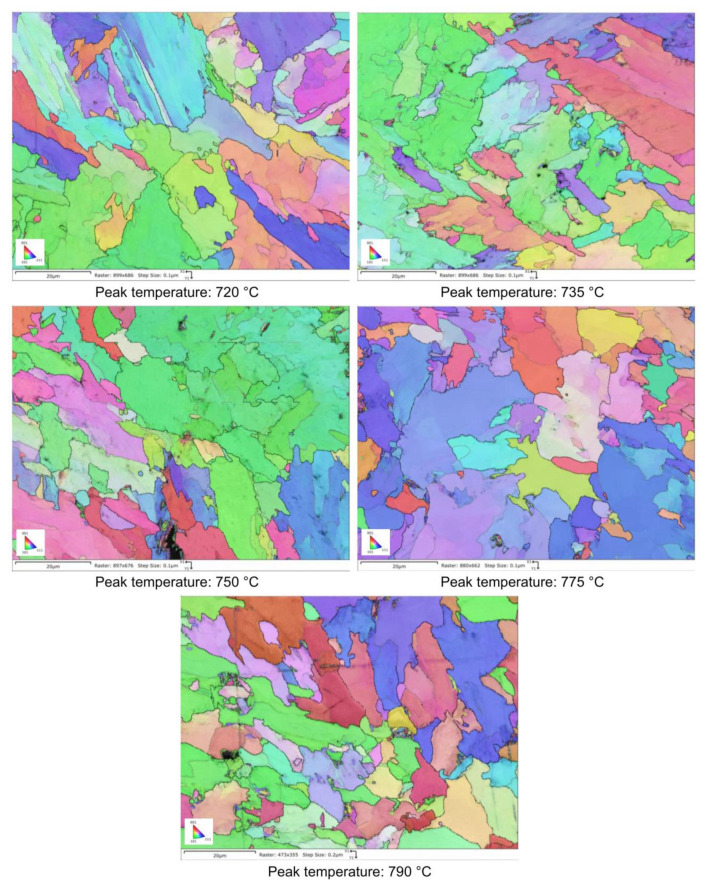
EBSD polar figure maps of specimens subjected to dilatometric cycles (variant I).

**Figure 9:**
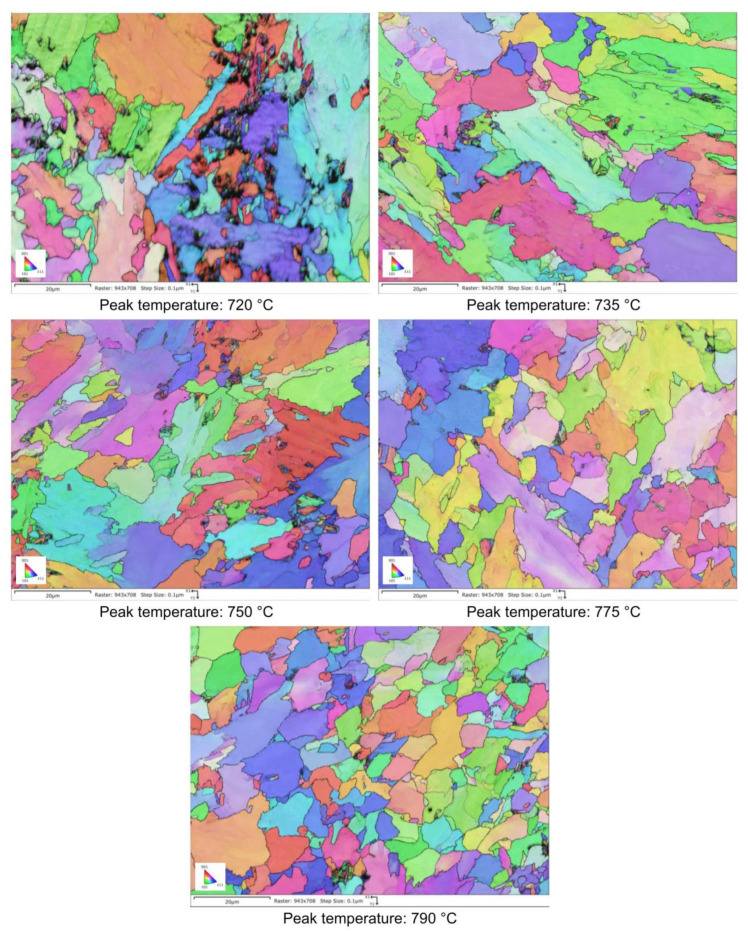
EBSD polar figure maps of specimens subjected to dilatometric cycles (variant II).

**Figure 10:**
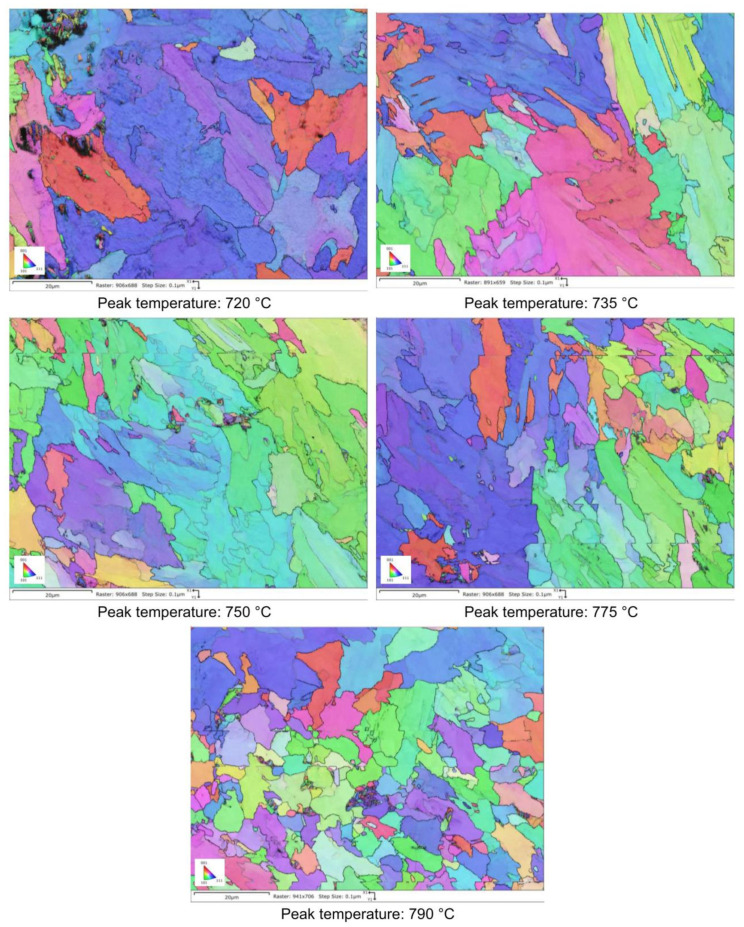
EBSD polar figure maps of specimens subjected to dilatometric cycles (variant III).

Moving from 790 °C to 720 °C the microstructure changes from ferrite-perlite to bainite, regardless the chemical composition, as confirmed by EBSD pole figure maps, reported in Figure 7, Figure 8, Figure 9 and Figure 10. This demonstrated that the formation of certain microstructural constituent, after welding thermal cycles, is not sensitive to micro-alloying addition in the selected ranges (V up to 0.10% and V-Nb addition up to 0.05%). Moreover, it is visible that Variant II shows the smallest and more uniform grain size among all the other variant for a peak temperature of 790 °C.

**Figure 11:**
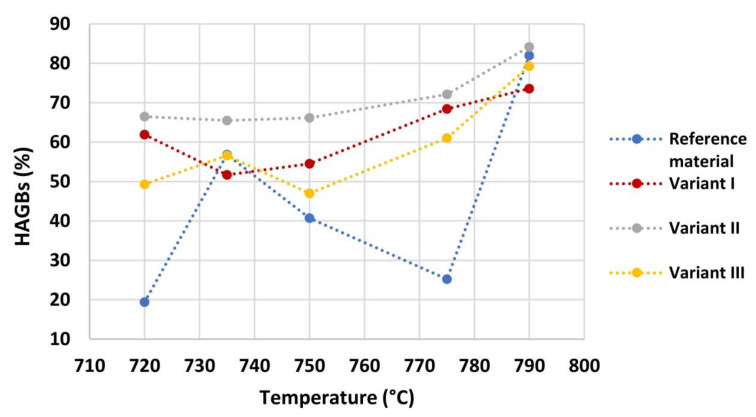
High-angle grain boundaries quantification (HAGBs %) (ϕ > 10°) for each condition.

Figure 11 shows the quantification of high angle grain boundaries (HAGBs %) (ϕ > 10°), obtained by EBSD analysis, for each condition. The tendency of all the variants, except for Reference material, is to increase HAGBs fraction with the increase of the inter-critical temperature. Variant II has the highest fraction of HAGBs in comparison to other variants. At the same time, Variant I shows the same trend of Variant II and exhibits higher fraction of HAGBs in comparison to Variant III, except for 735 °C and 790 °C. This suggests that, in regards to the HAGBs fraction, Variant II is expected to show the highest fatigue and toughness performance, since these grain boundaries type are responsible for a higher deflection of cracks during a fatigue cycle and an obstacle to cleavage propagation [44,45,46].

**Figure 12:**
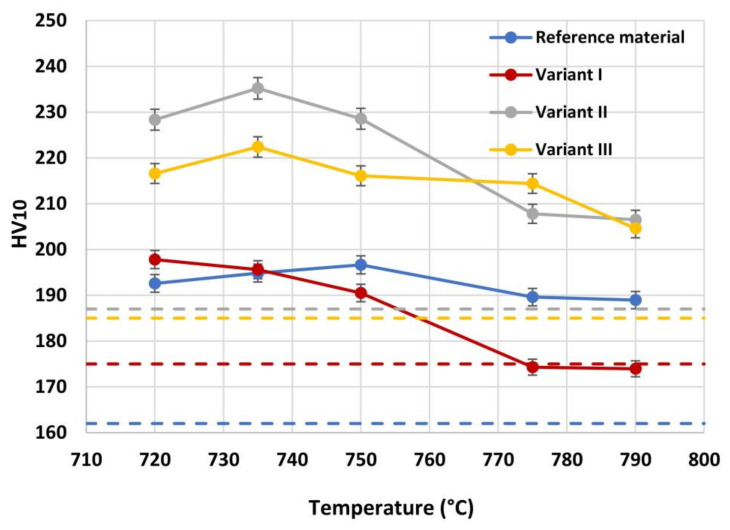
Hardness dependence on inter-critical temperature for the different considered materials.

The hardness dependence of all the considered variants as a function of the inter-critical temperature is reported in Figure 12. As expected, after welding thermal cycle, for each steel variant there is an increase of hardness value compared to hot rolled state. Moreover, while the reference material appears to be independent on the tested temperature, Variant I is subjected to a hardness loss starting from a hardness value approximately similar to that of the reference material. A clear effect of micro-alloying is reported for Variant II (0.10% V) and Variant III (0.03% V–0.02% Nb). These results show that an increase in the inter-critical temperature leads to a decrease of hardness. Typically, at a fixed inter-critical temperature of 720 °C an increase by approximately 30 Vickers points of each variant in comparison to its initial value in hot rolled state is present. For a temperature of 790 °C, Variant I kept the same hardness, Reference material experienced an increase of 30 Vickers points, while Variant III and Variant II experience an increase of hardness by 20 Vickers points. These results are consistent with the ones published by [47,48].

In particular, both for Variant II and for Variant III, a peak of hardness at 735 °C is evident and the nature of this behavior can be attributed to both the presence of residual austenite and a different precipitation state. A desired strengthening of the steels due to formation of the residual austenite would be detrimental in terms of toughness [30,31]. Otherwise, an adequate state of precipitation (fine and homogeneously dispersed precipitates) would ensure the strengthening and, at the same time, a better fatigue behavior [49]. The investigation of these aspects (RA and precipitation state) has been conducted and is illustrated in the Section 3.2 and Section 3.3.

### 3.2. Residual Austenite

**Figure 13:**
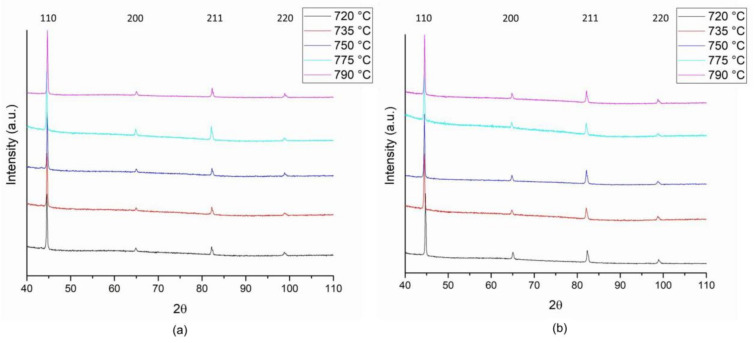
XRD spectra for reference material (**a**) and variant III (**b**) as a function of second temperature peak.

In order to evaluate the RA content on the above considered specimens, XRD patterns have been acquired. Results reported in Figure 13 for the reference material and for Variant III show no evidence of variation between the different conditions, since the RA fraction is low enough to stand below the intrinsic sensibility threshold of the technique (equal to about 1 wt.%). Therefore, X-ray diffraction technique showed to be not suitable for RA determination in HAZ for the considered set of samples.

**Figure 14:**
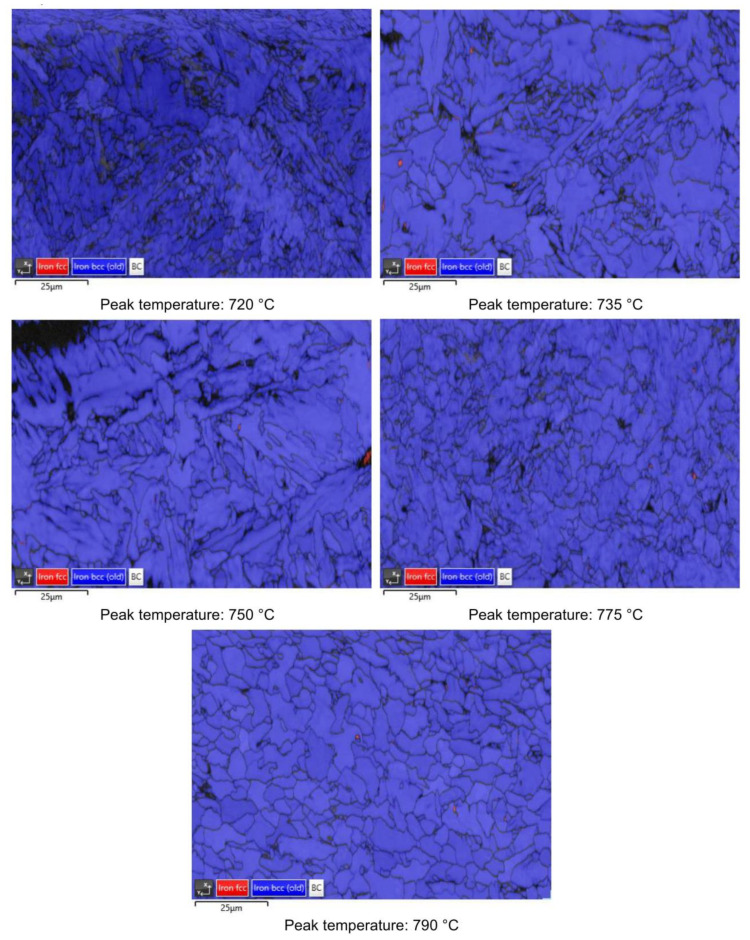
EBSD phase maps. Red zones: RA phase (reference material).

**Figure 15:**
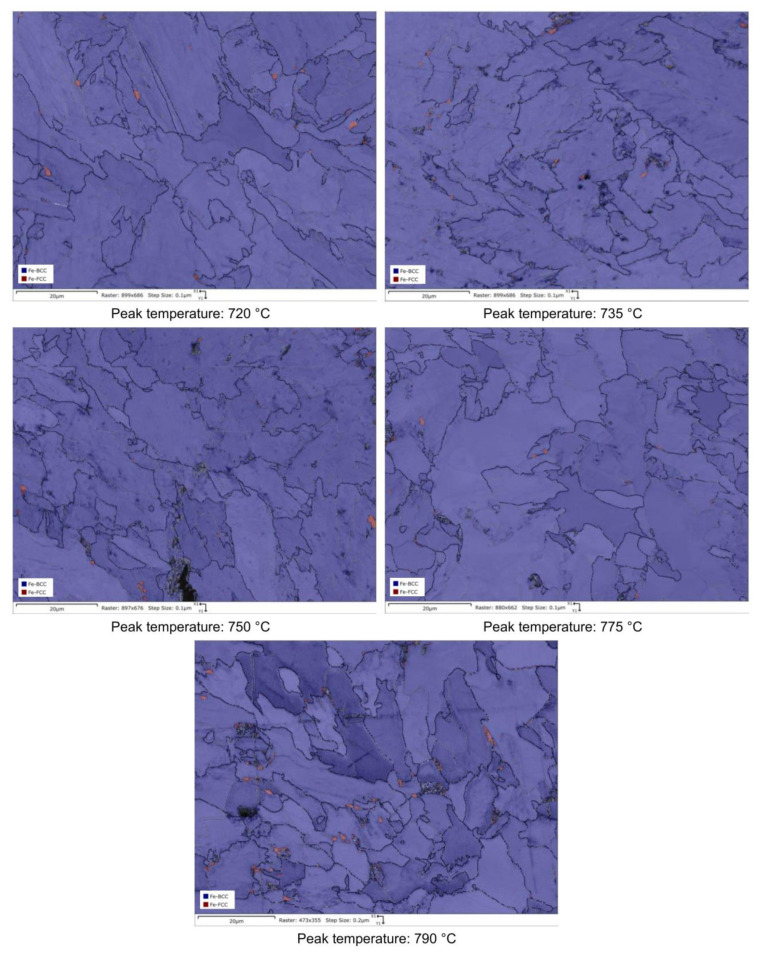
EBSD phase maps. Red zones: RA phase (variant I).

**Figure 16:**
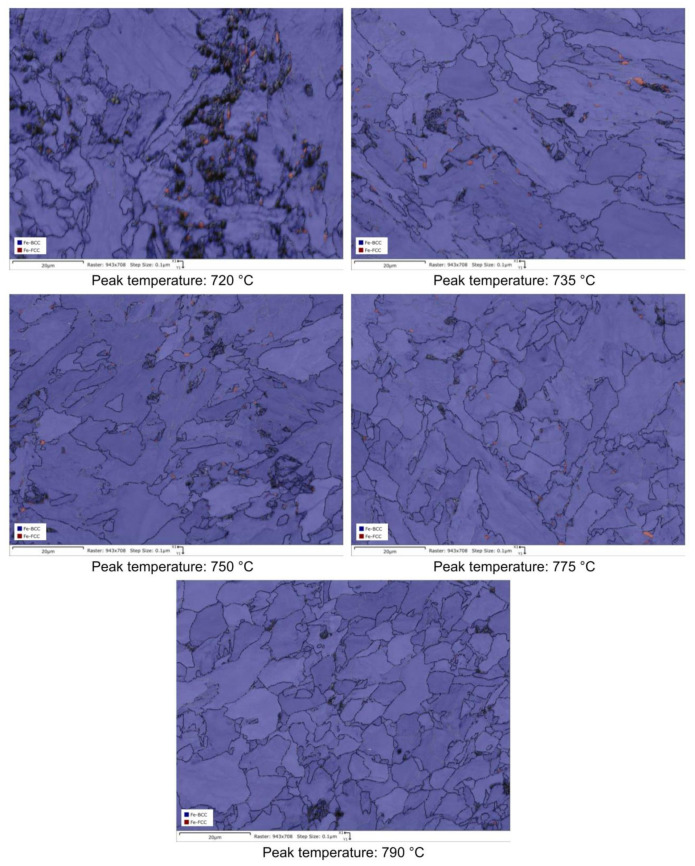
EBSD phase maps. Red zones: RA phase (variant II).

**Figure 17:**
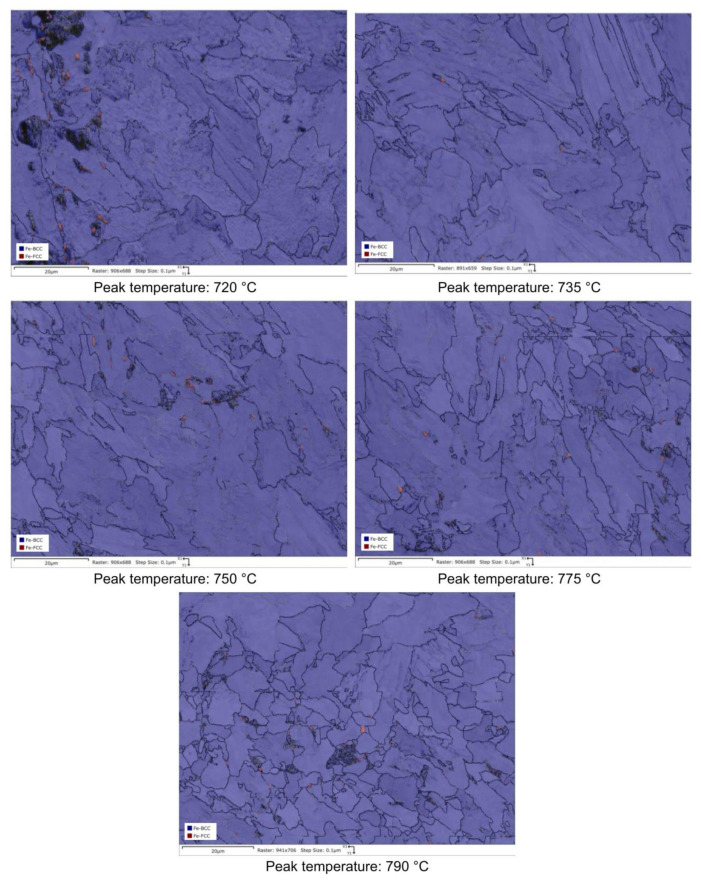
EBSD phase maps. Red zones: RA phase (variant III).

RA content has also been evaluated by the analysis of phase maps as obtained by EBSD (Figure 14, Figure 15, Figure 16 and Figure 17).

**Figure 18:**
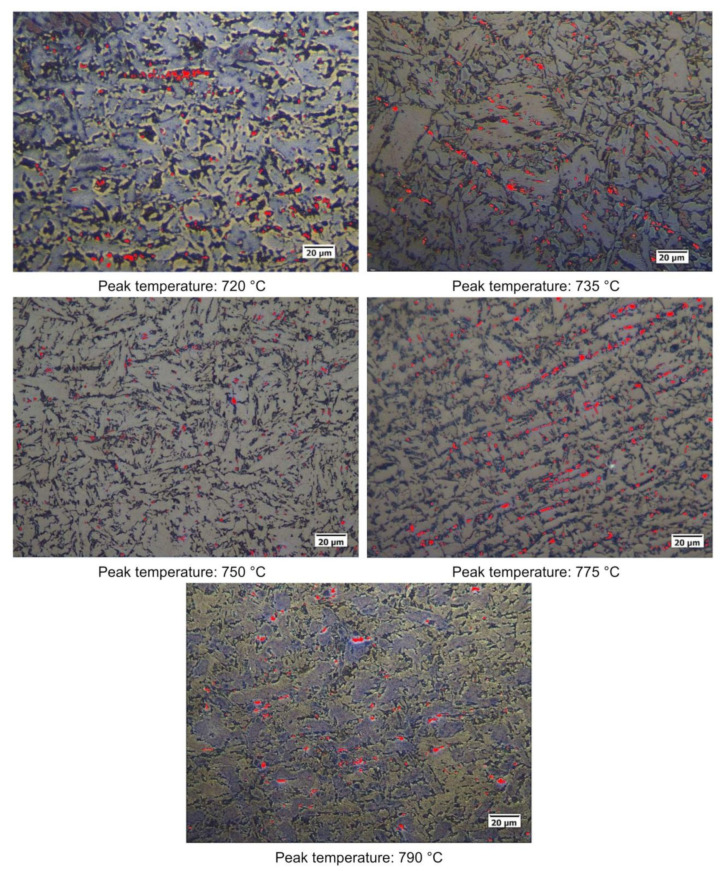
Microstructures of the considered specimens after LePerà etching. Red zones: RA phase (Variant I).

**Figure 19:**
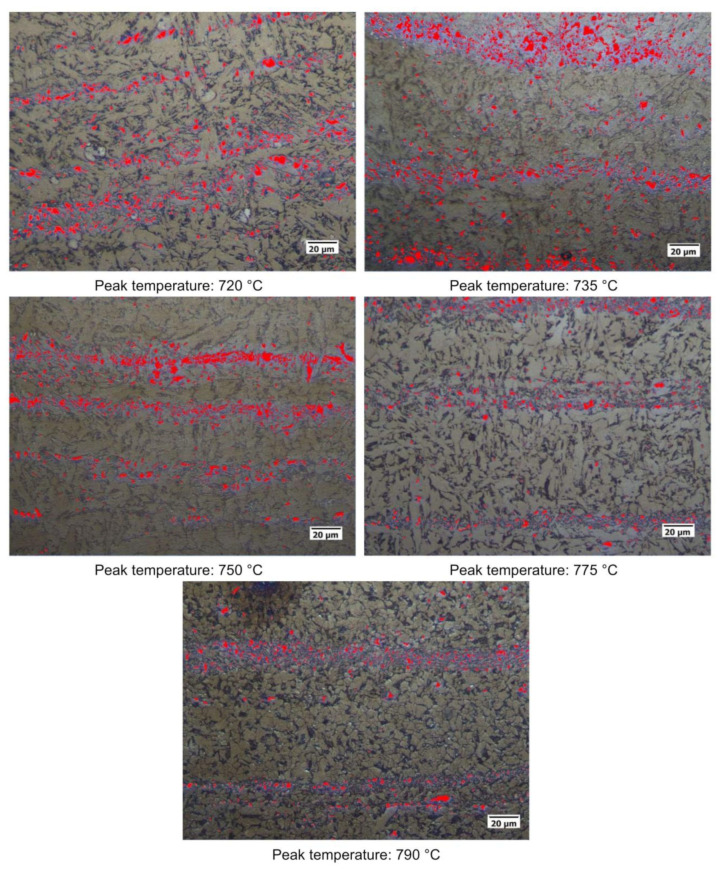
Microstructures of the considered specimens after LePerà etching. Red zones: RA phase (Variant II).

Quantitative optical microscopy metallography after LePerà selective etching has been applied to the same specimens and the micrographs referring to Variant I and Variant II are shown as an example in Figure 18 and Figure 19.

**Figure 20:**
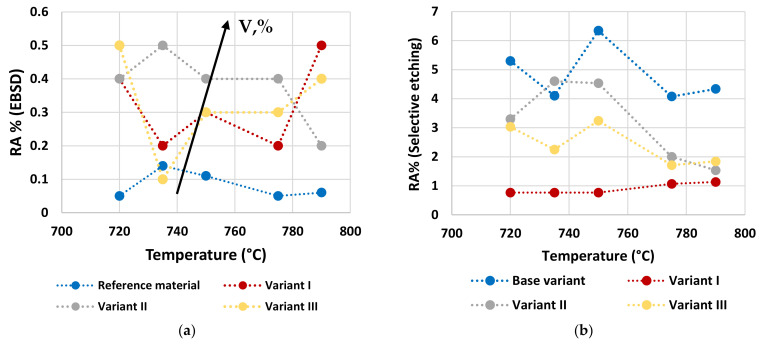
Quantified RA % with EBSD phase maps (**a**) and selective etching (**b**) as a function of the second peak temperature for the different considered steels.

RA fractions as a function of second peak temperature for the considered steels are reported in Figure 20, as obtained by the analysis of EBSD phase maps (Figure 20a) and by optical metallography after selective etching (Figure 20b), giving scope for a comparison. RA % values as obtained by light microscopy analysis after selective etching appears to be higher in magnitude than those by crystallographic information given by EBSD. Moreover, the reference material shows more residual austenite than the rest of the variants in the selective etching technique. Such a result, not predicted, is already mentioned in literature (e.g., [18]) and it is related to the fact that the selective etching could partially affect some zones neighboring the austenite areas, together with some limitations in the measurements accuracy. Therefore, although the EBSD technique applies for smaller investigation areas, it is still more accurate for evaluating RA % values compared to selective etching. As a consequence, the values obtained by analyzing the EBSD phase maps were considered more reliable. However, the capability of observing an extended area by optical microscopy enables the estimation of localization and distribution of residual austenite. In fact, as shown in Figure 18 and Figure 19, the constituent RA is arranged along bands, precisely in correspondence with the segregated areas of the original microstructure (Figure 1), where the stabilizing elements of austenite (such as C of Mn) are more concentrated [28]. It is also worth to mention that the RA values determined by means of EBSD technique are well below the X-ray diffraction threshold (equal to 1 wt.%), thus confirming the unsuitability of such a technique in the analysis of RA content in inter-critical zones of welded joints of considered steels. Furthermore, taking each steel variant as a separate reference, RA values shown in Figure 20 prove that the temperature of the second peak does not seem to influence the content of RA regardless of the technique used (either EBSD or LePerà selective etching). In this regard it is useful to consider these two methods as complementary, EBSD to determine the numerical value and LePerà selective etching method to give a localization overview of RA in HAZ of welded joint. Moreover, the experimental results of RA % obtained by EBSD technique (Figure 20a) show that V addition promotes the formation of residual austenite in agreement with [33]. However, there is no evidence that such low RA contents which were determined can be considered responsible for the hardness behavior reported in Figure 10. In this regard, in order to understand the hardness peak at 735 °C shown in Figure 10, the analysis of the precipitation state conducted by TEM is reported in the Section 3.3.

### 3.3. Precipitation State

**Figure 21:**
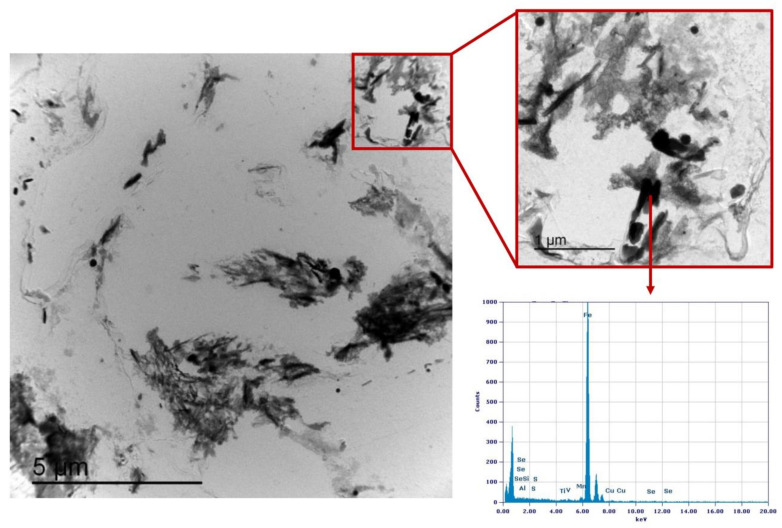
TEM micrograph of Variant II steel after inter-critical treatment with second peak temperature at 735 °C. Highlighted are the areas of cementite.

**Figure 22:**
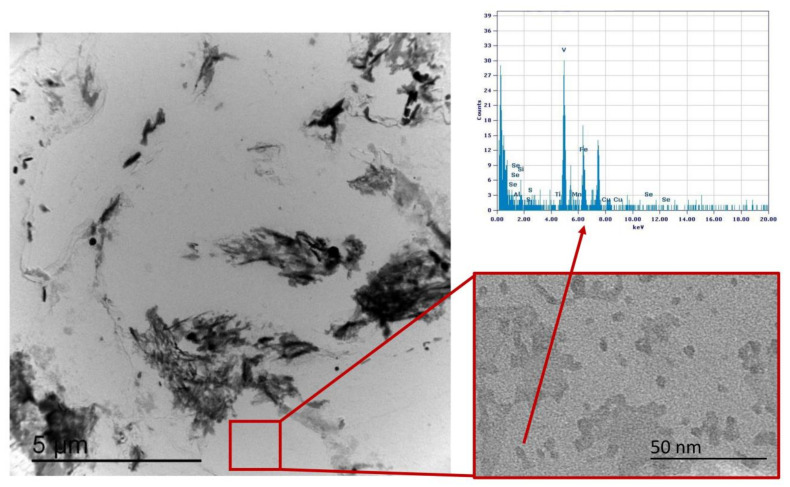
TEM micrograph of Variant II steel after inter-critical treatment with second peak temperature at 735 °C. Highlighted are the fine V-rich precipitates in the matrix.

**Figure 23:**
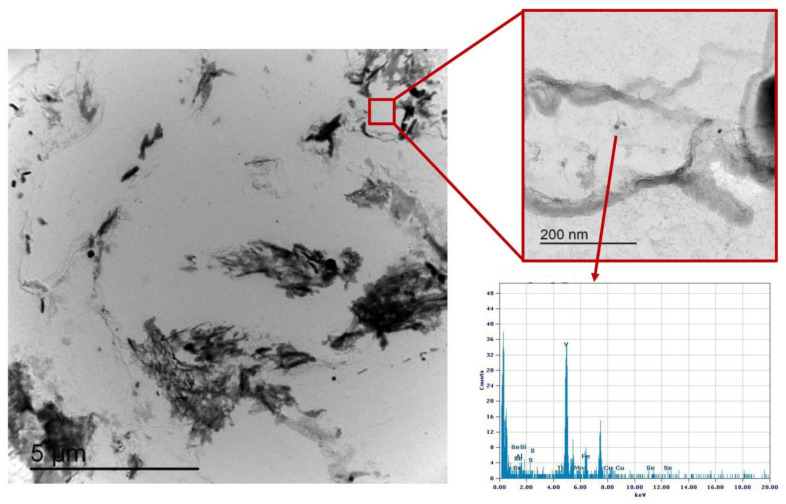
TEM micrograph detail of Variant II steel after inter-critical treatment with second peak temperature at 735 °C. Highlighted are the fine V-rich precipitates in the matrix.

**Figure 24:**
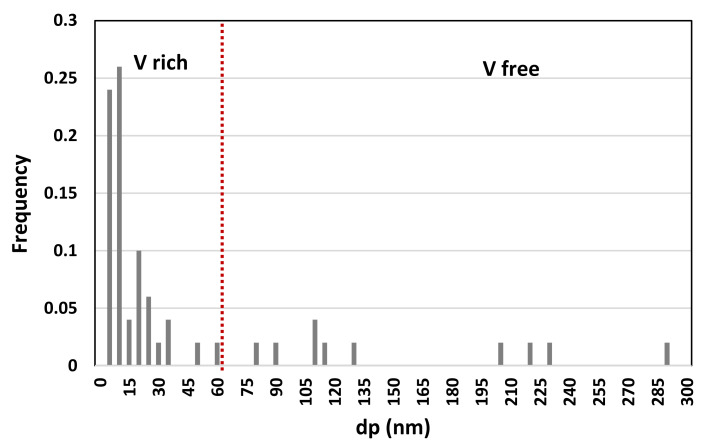
Precipitates size distribution (Variant II, 735 °C).

Precipitation state analysis has been performed on selected specimens corresponding to the highest hardness values, according to Figure 12, in order to focus on the most critical scenario on toughness and fatigue behavior perspective. In this paragraph, the analysis of the Variant II (0.10% V) and Variant III (0.03% V and 0.02% Nb) with second peak temperature in the inter-critical range at 735 °C is reported. Analysis of Variant II specimen shows the presence of cementite regions (as expected) (Figure 21) and others with very fine V-rich precipitates in the matrix (Figure 22 and Figure 23). The precipitates size distribution is reported in Figure 24, taking into account the chemical composition of precipitates. Results show that V-rich precipitates range sizes is below 60 nm (vanadium content in largest precipitates is lower than 0.5% as compared to a value larger than 30% for the precipitates smaller than 60 nm). In addition, more than 50% of V-rich precipitates have a size below 15 nm. This means that in such a condition vanadium addition does not appear to be critical in terms of fatigue resistance, as it would be expected in the case of its presence in largest precipitates [50].

**Figure 25:**
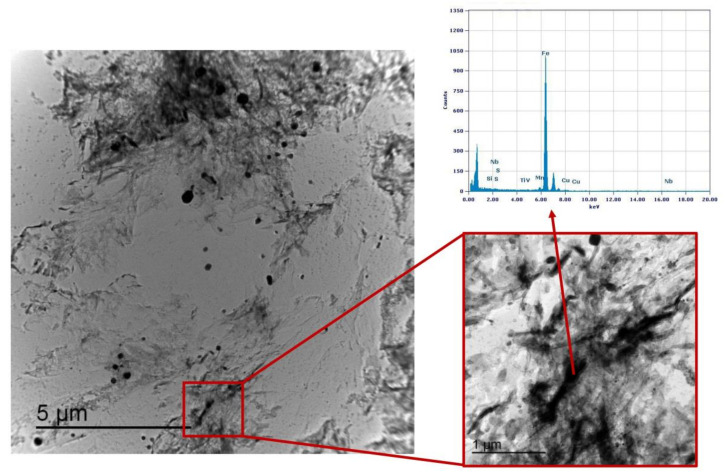
TEM micrograph of Variant III steel after inter-critical treatment with second peak temperature at 735 °C. Highlighted are the areas of cementite.

**Figure 26:**
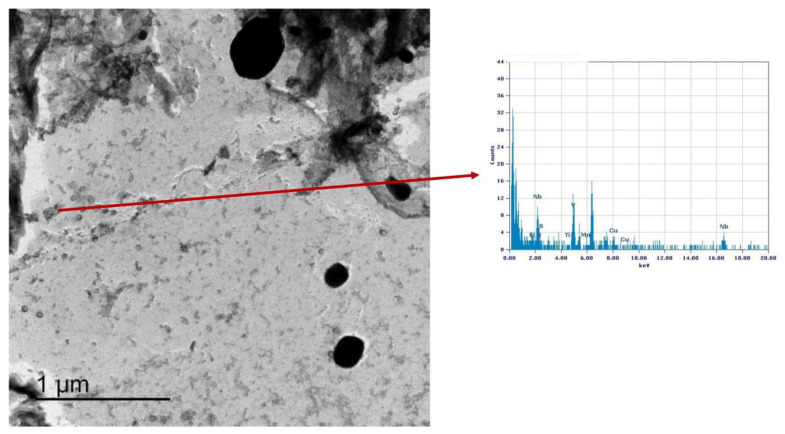
TEM micrograph of Variant III steel after inter-critical treatment with second peak temperature at 735 °C. Highlighted are the Nb-V rich precipitates in the matrix.

**Figure 27:**
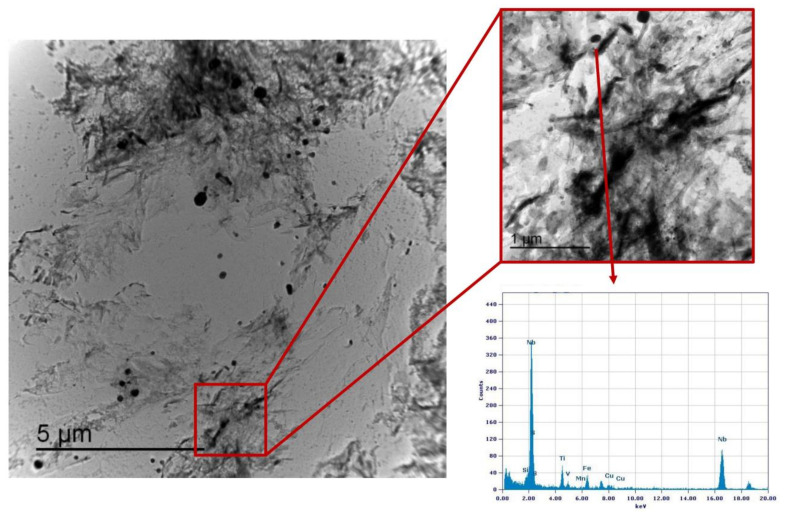
TEM micrograph of Variant III steel after inter-critical treatment with second peak temperature at 735 °C. Highlighted are the Nb rich precipitates in the matrix.

**Figure 28:**
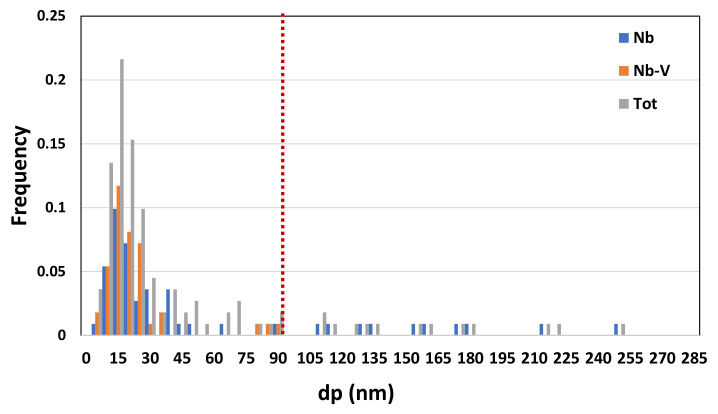
Precipitates size distribution (Variant III, 735 °C).

Similarly, the analysis of Variant III shows the presence of cementite (Figure 25) and areas with precipitates rich in Nb-V (Figure 26) and Nb (Figure 27). However, in this specific case, the distribution of the frequency of the size of the precipitates (Figure 28) shows a different behavior of the precipitation, compared to Variant II. V is always present combined with Nb, in precipitates smaller than 90 nm, whereas the larger precipitates, which size is up to 250 nm, are only rich in Nb. Furthermore, only 30% of the precipitates of Nb-V are smaller than 15 nm in size, evidencing that the combination of V and Nb micro-alloying could compromise the fatigue performance in the HAZ of a welded joint [51].

## 4. Conclusions

The behavior of the inter-critical region of a S355 grade steel with different vanadium content is reported in this paper. Double-pass welding thermal cycles were simulated using a dilatometer, with the maximum temperature of the secondary peak in the inter-critical area, in the range between 720 °C and 790 °C.

- Results show a negligible effect of vanadium addition on steel hardenability: this is consistent with a poor dependence of the microstructure type on steel chemical composition (in the considered variation range) taking into account the same inter-critical temperature.
- A low residual austenite value is found in combination with an increase of residual austenite content as the micro-alloying content increases. This result is independent on the inter-critical temperature and is consistent with the negligible effect on hardenability. EBSD technique has shown being more accurate in the quantification of RA fraction than selective etching in small areas, being consistent with them.
- HAGBs fraction, except for reference material, increase with the increase of inter-critical temperature for all the variants. The Variant II with high V content (0.1 wt.%) shows the highest HAGBs fraction for all the temperatures, which is expected to improve the fatigue and toughness behavior.
- Vanadium micro-alloying promotes the formation of very fine precipitates in the IC GC HAZ. Results also show that V-rich precipitates size is below 60 nm with more than 50% of V-rich precipitates having a size below 15 nm. This means that vanadium addition in such a condition does not appear to be critical in terms of fatigue resistance, as it would be expected in the case of its presence in largest precipitates. On the other hand, from the combination of V and Nb, the results of the analysis of the precipitates showed that V is present in precipitates smaller than 90 nm and combined with Nb and only 30% of the Nb-V precipitates have a size smaller than 15 nm. Furthermore, the precipitates with size larger than 90 nm are purely Nb, which is compromising for fatigue behavior.

In conclusion, in this work it has been demonstrated that vanadium addition in HSLA steel does not lead to the formation of a significant percentage of residual austenite in IC GC HAZ of a double-pass welding process. The combination of a more fine-grained microstructure, higher fraction of HAGBs and the formation of fine precipitates, can be promising for the improvement of fatigue and toughness behavior. This makes the adoption of high strength vanadium micro-alloyed steels very promising in structural applications, also enabling the use of a reduced quantity of raw-materials, hence mitigating the environmental impact of the resulting formulations.

## Figures and Tables

**Table 1:** Nominal chemical composition of the considered steels (wt.%) (Fe to balance).

	C	Mn	V	Si	Nb
Reference material	0.16	1.45	-	0.03	-
Variant I	0.16	1.45	0.05	0.03	-
Variant II	0.16	1.45	0.10	0.03	-
Variant III	0.16	1.45	0.03	0.03	0.02

**Table 2:** Critical temperature Ac1 and Ac3 evaluated by means of dilatometric test.

	Ac1 (°C)	Ac3 (°C)
Reference material	715	825
Variant I	712	820
Variant II	715	845
Variant III	700	815

## Data Availability

The data presented in this study are available on request from the corresponding author.

## References

[1] Kim D.W., Yang J., Kim Y.G., Kim W.K., Lee S., Sohn S.S. (2022). Effects of Granular Bainite and Polygonal Ferrite on Yield Strength Anisotropy in API X65 Linepipe Steel. Mater. Sci. Eng. A.

[2] Roy S., Romualdi N., Yamada K., Poole W., Militzer M., Collins L. (2022). The Relationship Between Microstructure and Hardness in the Heat-Affected Zone of Line Pipe Steels. JOM.

[3] Stornelli G., Di Schino A., Mancini S., Montanari R., Testani C., Varone A. (2021). Grain Refinement and Improved Mechanical Properties of Eurofer97 by Thermo-Mechanical Treatments. Appl. Sci..

[4] Stornelli G., Gaggia D., Rallini M., Di Schino A. (2021). Heat Treatment Effect on Maraging Steel Manufactured by Laser Powder Bed Fusion Technology: Microstructure and Mechanical Properties. Acta Metall. Slovaca.

[5] Stornelli G., Gaggiotti M., Mancini S., Napoli G., Rocchi C., Tirasso C., Di Schino A. (2022). Recrystallization and Grain Growth of AISI 904L Super-Austenitic Stainless Steel: A Multivariate Regression Approach. Metals.

[6] Lo K.H., Shek C.H., Lai J.K.L. (2009). Recent Developments in Stainless Steels. Mater. Sci. Eng. R Rep..

[7] Benz J. (2017). The Effect of Vanadium and Other Microalloying Elements on the Microstructure and Properties of Bainitic HSLA Steels. Mater. Sci. Technol. Conf. Exhib..

[8] Fazeli F., Amirkhiz B.S., Scott C., Arafin M., Collins L. (2018). Kinetics and Microstructural Change of Low-Carbon Bainite Due to Vanadium Microalloying. Mater. Sci. Eng. A.

[9] Baker T.N. (2016). Microalloyed Steels. Ironmak. Steelmak..

[10] Di Schino A., Gaggiotti M., Testani C. (2020). Heat Treatment Effect on Microstructure Evolution in a 7% Cr Steel for Forging. Metals.

[11] Tian Y., Zhao M.C., Zeng Y.P., Shi X.B., Yan W., Yang K., Zeng T.Y. (2022). Elimination of Primary NbC Carbides in HSLA Steels for Oil Industry Tubular Goods. JOM.

[12] Li X., Cai Z., Hu M., Li K., Hou M., Pan J. (2021). Effect of NbC Precipitation on Toughness of X12CrMoWNbVN10-1-1 Martensitic Heat Resistant Steel for Steam Turbine Blade. J. Mater. Res. Technol..

[13] Mancini S., Langellotto L., Di Nunzio P.E., Zitelli C., Di Schino A. (2020). Defect Reduction and Quality Optimization by Modeling Plastic Deformation and Metallurgical Evolution in Ferritic Stainless Steels. Metals.

[14] Bay Y., Bhattacharyya R., Mc Cormick M.E. (2001). Use of High Strength Steels. Elsevier Ocean. Eng. Ser..

[15] Narimani M., Hajjari E., Eskandari M., Szpunar J.A. (2022). Electron Backscattered Diffraction Characterization of S900 HSLA Steel Welded Joints and Evolution of Mechanical Properties. J. Mater. Eng. Perform..

[16] Geng R., Li J., Shi C., Zhi J., Lu B. (2022). Effect of Ce on Microstructures, Carbides and Mechanical Properties in Simulated Coarse-Grained Heat-Affected Zone of 800-MPa High-Strength Low-Alloy Steel. Mater. Sci. Eng. A.

[17] Shi S.C., Wang W.C., Ko D.K. (2022). Influence of Inclusions on Mechanical Properties in Flash Butt Welding Joint of High-Strength Low-Alloy Steel. Metals.

[18] Lambert-Perlade A., Gourgues A.F., Besson J., Sturel T., Pineau A. (2004). Mechanisms and Modeling of Cleavage Fracture in Simulated Heat-Affected Zone Microstructures of a High-Strength Low Alloy Steel. Metall. Mater. Trans. A.

[19] Miletić I., Ilić A., Nikolić R.R., Ulewicz R., Ivanović L., Sczygiol N. (2020). Analysis of Selected Properties of Welded Joints of the HSLA Steels. Materials.

[20] Cho L., Tselikova A., Holtgrewe K., de Moor E., Schmidt R., Findley K.O. (2022). Critical Assessment 42: Acicular Ferrite Formation and Its Influence on Weld Metal and Heat-Affected Zone Properties of Steels. Mater. Sci. Technol..

[21] Kim B.C., Lee S., Kim N.J., Lee D.Y. (1991). Microstructure and Local Brittle Zone phenomena in High-Strength Low-Alloy Steel Welds. Metall. Trans. A.

[22] Di Schino A., Mortello M., Schmidt R., Stornelli g., Tselikiva A., Zucca G. (2023). Studio della microstruttura in zona termicamente alterata di un acciaio S355 microlegato al vanadio. Riv. Ital. Della Saldatura.

[23] Prasad K., Dwivedi D.K. (2008). Some Investigations on Microstructure and Mechanical Properties of Submerged Arc Welded HSLA Steel Joints. Int. J. Adv. Manuf. Technol..

[24] Kvackaj T., Bidulská J., Bidulský R. (2021). Overview of Hss Steel Grades Development and Study of Reheating Condition Effects on Austenite Grain Size Changes. Materials.

[25] Mengaroni S., Cianetti F., Calderini M., Evangelista E., Di Schino A., McQueen H. (2015). Tool steels: Forging simulation and microstructure evolution of large-scale ingot. Acta Phys. Pol. A.

[26] Ouchi C. (2001). Advances in Physical Metallurgy and Processing of Steels. Development of Steel Plates by Intensive Use of TMCP and Direct Quenching Processes. ISIJ Int..

[27] Spanos G., Fonda R.W., Vandermeer R.A., Matuszeski A. (1995). Microstructural Changes in HSLA-100 Steel Thermally Cycled to Simulate the Heat-Affected Zone during Welding. Metall. Mater. Trans. A.

[28] Di Schino A., Testani C. (2020). Corrosion behavior and mechanical properties of AISI 316 stainless steel clad Q235 plate. Metals.

[29] Lee S., Kim B.C., Kwon D. (1992). Correlation of Microstructure and Fracture Properties in Weld Heat- Affected Zones of Thermomechanically Controlled Processed Steels. Metall. Trans. A.

[30] Taillard R., Verrier P., Maurickx T., Foct J. (1995). Effect of Silicon on CGHAZ Toughness and Microstructure of Microalloyed Steels. Metall. Mater. Trans. A.

[31] Taillard R., Foct J., Verrier P., Maurickx T. (1989). Residual Austenite and Its Effect on Fracture Toughness of Coarse-Grained Heat-Affected Zone of H.S.L.A. Steels. Proceedings of the ESOMAT 1989—Ist European Symposium on Martensitic Transformations in Science and Technology.

[32] Cui J., Zhu W., Chen Z., Chen L. (2020). Microstructural Characteristics and Impact Fracture Behaviors of a Novel High-Strength Low-Carbon Bainitic Steel with Different Reheated Coarse-Grained Heat-Affected Zones. Met. Mater. Trans. A Phys. Met. Mater. Sci..

[33] Li Y., Crowther D.N., Green M.J.W., Mitchell P.S., Baker T.N. (2001). The Effect of Vanadium and Niobium on the Properties and Microstructure of the Intercritically Reheated Coarse Grained Heat Affected Zone in Low Carbon Microalloyed Steels. ISIJ Int..

[34] Mitchell P.S., Hart P.H.M., Morrison W.B. The Effect of Microalloying on HAZ Toughness. Proceedings of the International Conference Microalloying.

[35] Babu S.S., Bhadeshia H.K.D.H. (1992). Stress and the Acicular Ferrite Transformation. Mater. Sci. Eng. A.

[36] Stornelli G., Gaggiotti M., Gattia D.M., Schmidt R., Sgambetterra M., Tselikova A., Zucca G., Di Schino A. (2022). Vanadium alloying in S355 structural steel: Effect on residual austenite formation in welded joints heat affected zone. Acta Metall. Slovaca.

[37] Zajac S., Siwecki T., Hutchinson B., Svensson L.E., Attlegård M. (1991). Weldability of High Nitrogen Ti-V Microalloyed Steel Plates Processed via Thermomechanical Controlled Rolling.

[38] Hu J., Du L.X., Wang J.J., Xie H., Gao C.R., Misra R.D.K. (2014). High Toughness in the Intercritically Reheated Coarse-Grained (ICRCG) Heat-Affected Zone (HAZ) of Low Carbon Microalloyed Steel. Mater. Sci. Eng. A.

[39] Wu H., Xia D., Ma H., Du Y., Gao C., Gao X., Du L. (2022). Study on Microstructure Characterization and Impact Toughness in the Reheated Coarse-Grained Heat Affected Zone of V-N Microalloyed Steel. J. Mater. Eng. Perform..

[40] Kasuya T., Yurioka N. (1993). Carbon Equivalent and Multiplying Factor for Hardenability of Steel. Weld. Res. Suppl..

[41] Li Y., Baker T.N. (2010). Effect of Morphology of Martensite–Austenite Phase on Fracture of Weld Heat Affected Zone in Vanadium and Niobium Microalloyed Steels. Mater. Sci. Technol..

[42] Qi X., Di H., Wang X., Liu Z., Misra R.D.K., Huan P., Gao Y. (2020). Effect of Secondary Peak Temperature on Microstructure and Toughness in ICCGHAZ of Laser-Arc Hybrid Welded X100 Pipeline Steel Joints. J. Mater. Res. Technol..

[43] Sun J., Hensel J., Klassen J., Nitschke-Pagel T., Dilger K. (2019). Solid-State Phase Transformation and Strain Hardening on the Residual Stresses in S355 Steel Weldments. J. Mater. Process. Technol..

[44] Díaz-fuentes M., Iza-mendia A., Gutiérrez I. (2003). Analysis of Different Acicular Ferrite Microstructures in Low-Carbon Steels by Electron Backscattered Diffraction. Study of Their Toughness Behavior. Metall. Mater. Trans. A.

[45] Zhao M.C., Yang K., Shan Y.Y. (2003). Comparison on Strength and Toughness Behaviors of Microalloyed Pipeline Steels with Acicular Ferrite and Ultrafine Ferrite. Mater. Lett..

[46] Rodriguez-Ibabe J.M. (1998). The Role of Microstructure in Toughness Behaviour of Microalloyed Steels. Mater. Sci. Forum.

[47] Zhu Z., Kuzmikova L., Li H., Barbaro F. (2014). Effect of Inter-Critically Reheating Temperature on Microstructure and Properties of Simulated Inter-Critically Reheated Coarse Grained Heat Affected Zone in X70 Steel. Mater. Sci. Eng. A.

[48] Moeinifar S., Kokabi A.H., Madaah Hosseini H.R. (2010). Influence of Peak Temperature during Simulation and Real Thermal Cycles on Microstructure and Fracture Properties of the Reheated Zones. Mater. Des..

[49] Hajisafari M., Nategh S., Yoozbashizadeh H., Ekrami A. (2013). Fatigue Properties of Heat-Treated 30MSV6 Vanadium Microalloyed Steel. J. Mater. Eng. Perform..

[50] Avtokratova E., Sitdikov O., Latypova O.E., Markushev M.v., Linderov M.L., Merson D.L., Vinogradov Y.A. (2018). Effect of Precipitates on Static and Fatigue Strength of a Severely Forged Aluminum Alloy 1570C. Proceedings of the IOP Conference Series: Materials Science and Engineering.

[51] Di Schino A., Barteri M., Kenny J.M. (2003). Fatigue behavior of a high nitrogen austenitic stainless steel as a function of its grain size. J. Mater. Sci. Lett..

